# The role and targeting strategies of non-coding RNAs in immunotherapy resistance in oral squamous cell carcinoma

**DOI:** 10.3389/fcell.2025.1642417

**Published:** 2025-10-27

**Authors:** Zhinan Liang, Yicheng Zhao, Xin Wang, Yuehe Li

**Affiliations:** ^1^ Department of Radiation Oncology, Fengcheng Hospital of Fengxian District, Shanghai, China; ^2^ Clinical Medical College, Yangzhou University, Yangzhou, China; ^3^ Key Laboratory of Systems Health Science of Zhejiang Province, School of Life Science, Hangzhou Institute for Advanced Study, University of Chinese Academy of Sciences, Hangzhou, China

**Keywords:** oral squamous cell carcinoma (OSCC), non-coding RNA (ncRNA), immunotherapy resistance, tumor microenvironment, targeting strategies

## Abstract

Oral squamous cell carcinoma (OSCC) represents a major global health burden, with resistance to immune checkpoint inhibitors (ICIs) posing a significant barrier to effective immunotherapy. Emerging evidence implicates non-coding RNAs (ncRNAs)—including microRNAs (miRNAs), long non-coding RNAs (lncRNAs), and circular RNAs (circRNAs)—as pivotal regulators of this resistance. In this review, we discuss how ncRNAs contribute to OSCC immunotherapy resistance by modulating immune checkpoint expression, suppressing anti-tumor T cell function while promoting immunosuppressive Tregs, reprogramming the tumor microenvironment (TME) via metabolic remodeling and myeloid cell regulation, and enhancing intrinsic tumor resistance through epigenetic alterations and cancer stem cell activation. These multifaceted roles highlight the therapeutic potential of targeting ncRNAs. Strategies involve inhibiting oncogenic ncRNAs or restoring tumor-suppressive counterparts, facilitated by advanced delivery methods like nanoparticles or exosomes. Combining ncRNA-based therapies with ICIs offers a promising approach to overcome resistance. Key challenges remain, including precise functional annotation, efficient and specific delivery, experimental validation, biomarker identification, and the design of optimized clinical trials—potentially guided by artificial intelligence and multi-omics approaches. Ultimately, targeting the complex ncRNA networks may offer transformative improvements in immunotherapy outcomes for OSCC patients.

## 1 Introduction

Oral cancer constitutes a major global health challenge, with oral squamous cell carcinoma (OSCC) being the predominant histological type, accounting for approximately 90% of cases (Chai et al., 2020; Kujan et al., 2020). In 2022 alone, there were an estimated 389,485 new cases and 188,230 deaths worldwide (Bray et al., 2024). Despite advances in treatment modalities, the overall prognosis for oral cancer patients remains suboptimal. The average 5-year survival rate hovers around 50%–60% (Lu et al., 2024), a figure that has seen little significant improvement over recent decades (Shao et al., 2025).

The advent of immunotherapy, particularly immune checkpoint inhibitors (ICIs), has marked a significant shift in cancer treatment, including for OSCC (Ma and Kim, 2024). ICIs, such as antibodies targeting programmed cell death protein 1 (PD-1) or its ligand PD-L1, and cytotoxic T-lymphocyte-associated protein 4 (CTLA-4), function by releasing the brakes on the host immune system, enabling T cells to recognize and attack tumor cells (Mivehchi et al., 2025). The expression of PD-L1 in tumor samples has shown potential as a prognostic and predictive biomarker, supporting the use of ICIs in OSCC management. However, immunotherapy benefits only a subset of OSCC patients. A substantial proportion of patients exhibit primary resistance (lack of initial response) or develop acquired resistance after an initial period of response (Chen F. et al., 2023). The overall response rates to ICI monotherapy in OSCC 10%–20%, benefiting only a fraction of patients (Ries et al., 2024). This limited efficacy highlights significant gaps in our understanding of the complex interactions between the tumor, its microenvironment, and the host immune system that dictate treatment success or failure (He et al., 2025). The heterogeneity of OSCC and the intricate mechanisms enabling tumor cells to evade immune surveillance are major obstacles (Wickenhauser et al., 2021). Therefore, elucidating the mechanisms underlying immune escape and immunotherapy resistance is paramount to developing strategies that can broaden the applicability and enhance the durability of immunotherapeutic responses in oral cancer patients (Li S. et al., 2024).

Non-coding RNAs (ncRNAs) are recognized as crucial functional molecules involved in virtually all aspects of cellular biology, with their dysregulation linked to various human diseases, including cancer (Anastasiadou et al., 2018). Aberrant ncRNA expression can drive tumorigenesis, promote metastasis, and contribute to therapy resistance by acting as oncogenic ncRNAs (oncomiRs, onco-lncRNAs) (Zhu P. et al., 2023). They participate in modulating immune checkpoint pathways and shaping the tumor microenvironment (TME) (Denaro et al., 2019). In the context of oral cancer, dysregulated expression of various miRNAs, lncRNAs, and circRNAs has been linked to disease initiation, progression, metastasis, and resistance to therapies like chemotherapy (Liu H. et al., 2022). This convergence of ncRNA involvement in both cancer biology and immune regulation strongly suggests their pivotal role in mediating the immune escape mechanisms observed in the oral cancer TME (Dey et al., 2023). Understanding these roles is critical, as ncRNAs represent a largely untapped source of potential diagnostic biomarkers and therapeutic targets to overcome immunotherapy limitations (Li R. et al., 2024; Fan et al., 2025).

This review systematically delineates the mechanisms by which ncRNAs, particularly miRNAs, lncRNAs, and circRNAs, mediate immunotherapy resistance in OSCC, with a focus on their regulation of key immune processes within the OSCC TME. We highlight the intricate molecular mechanisms by which ncRNAs modulate immune checkpoint expression, reshape the immune landscape, and promote a suppressive TME. In parallel, we discuss emerging therapeutic strategies that target these ncRNAs, including both inhibitory and restorative approaches, and explore their potential when combined with ICIs. Finally, we address the key challenges that hinder the clinical translation of ncRNA-based diagnostics and therapeutics, while outlining promising directions for future research aimed at overcoming these barriers.

## 2 Core mechanisms of ncRNA-Mediated immunotherapy resistance

ncRNAs are broadly classified based on size, with small ncRNAs (<200 nucleotides) including microRNAs (miRNAs), small interfering RNAs (siRNAs), and Piwi-interacting RNAs (piRNAs), and long non-coding RNAs (lncRNAs) comprising transcripts >200 nucleotides (Mehler and Mattick, 2007). Circular RNAs (circRNAs), characterized by a covalently closed loop structure, represent another major class (Kos et al., 1986). ncRNAs exert their regulatory functions through diverse and intricate mechanisms (Chen K. et al., 2023). miRNAs typically function post-transcriptionally by binding to target messenger RNAs (mRNAs), primarily within the 3′ untranslated region (UTR), leading to mRNA degradation or translational repression (Moore, 2005). LncRNAs exhibit remarkable functional versatility; they can act as molecular scaffolds assembling protein complexes, as guides recruiting chromatin-modifying enzymes (like Polycomb Repressive Complex 2, PRC2) to specific genomic loci for epigenetic regulation (Balas et al., 2021), as decoys sequestering proteins or miRNAs, and as competing endogenous RNAs (ceRNAs) that “sponge” miRNAs, thereby regulating the expression of other miRNA targets (Salmena et al., 2011). CircRNAs, owing to their stability, primarily function as potent miRNA sponges but can also interact with proteins and regulate transcription or splicing (Memczak et al., 2013).

**FIGURE 1:**
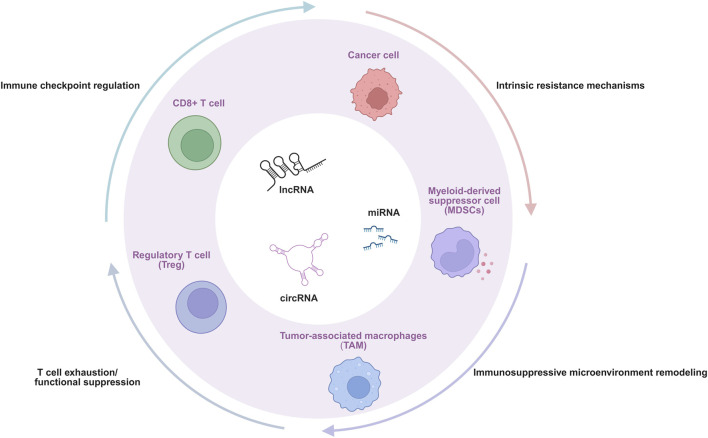
Core mechanisms of ncRNA-mediated immunotherapy resistance.

Given the indispensable role of ncRNAs in gene expression regulatory networks and their broad impact on various tumor biological behaviors, their involvement in the complex process of immune escape in oral cancer is receiving increasing attention (Memczak et al., 2013; Chen et al., 2017; O'Connell et al., 2010). ncRNAs do not function via a single pathway; instead, they weave a multi-level, multi-faceted regulatory network, intricately manipulating the interaction between tumor cells and the immune system, ultimately leading to the failure of immune surveillance and tumor progression (Denaro et al., 2019; O'Connell et al., 2010). Although numerous pathways are involved, current research evidence and therapeutic relevance increasingly point to four interconnected and crucial core mechanisms that constitute the main pillars of ncRNA-mediated immune escape in oral cancer. These four core mechanisms are: (Chai et al., 2020): the direct regulation of key immune checkpoint molecules, which affects T cell activation and tumor immune visibility (Saadi et al., 2022; Kujan et al., 2020) the induction of T cell exhaustion and functional suppression, alongside the promotion of regulatory T cells (Tregs) (Li et al., 2025; Afra et al., 2024); (Bray et al., 2024) the profound remodeling of the immunosuppressive TME, including influencing metabolic reprogramming and myeloid cell populations; and (Lu et al., 2024) the enhancement of intrinsic resistance mechanisms within tumor cells. These interconnected processes collectively enable tumor immune evasion (Figure 1). Following we will dissect in detail how various types of ncRNAs (miRNAs, lncRNAs, and circRNAs) specifically mediate immune escape in oral cancer through these four core mechanisms (Table 1).

**TABLE 1:** Mechanisms of ncRNA-Mediated Immunotherapy Resistance in OSCC.

Mechanism category	ncRNA type	Specific ncRNA	Specific effect/Key target/Mechanism	References(s)
Immune Checkpoint Regulation	miRNA	miR-197	Downregulates PD-L1 (Indirectly suggested via CKS1B/STAT3 pathway)	Fujita et al. (2015), Thomas et al. (2024), Jiang et al. (2024)
	miRNA	miR-495-3p	Downregulates PD-L1 (Direct targeting; Sequestered by CircKRT1)	Yang et al. (2021)
	miRNA	miR-876-5p	Upregulates PD-L1 (Targets SOCS4 - > activates STAT3)	Peng et al. (2025)
	lncRNA	IFITM4P	Upregulates PD-L1 (Cytoplasm: NF-κB activation; Nucleus: KDM5A recruitment inhibits PTEN - > PI3K/AKT activation)	Shi et al. (2022)
	lncRNA	MALAT1	Upregulates PD-L1 (Sponges miR-200 family; Potential EZH2 interaction; Potential m6A stabilization)	Xu et al. (2024), Cheng et al. (2018), Wang et al. (2015), Song et al. (2022)
	lncRNA	HOTAIR	Upregulates PD-L1 (Mechanisms like miR-sponging, NF-κB activation, EV transfer shown in other cancers)	Yuan et al. (2022), Wang et al. (2021), Tuersong et al. (2025)
	circRNA	CircKRT1	Upregulates PD-L1 (Sponges miR-495-3p)	Yang et al. (2021), Zhu et al. (2023b)
	circRNA	hsa_circ_0069313	Upregulates PD-L1 (Sponges miR-325-3p - > increases Foxp3)	Chen et al. (2022b)
T Cell Dysfunction (Exhaustion & Treg Function)	lncRNA	CRNDE	Promotes CD8^+^ T cell exhaustion (Sponges miR-545-5p - > upregulates TIM-3)	Ai SW et al. (2024), Ai et al. (2021)
	lncRNA	Lnc-Tim3	Promotes CD8^+^ T cell exhaustion (Binds TIM-3 protein, modulates signaling - HCC context)	Ji et al. (2018)
	circRNA	hsa_circ_0069313	Enhances Treg immunosuppression (Sponges miR-325-3p - > increases Foxp3 in tumor cells and via exosomes in Tregs)	Chen et al. (2022b)
	circRNA	circQSOX1	Induces Tregs, contributes to anti-CTLA-4 resistance	Liu et al. (2022b)
TME Remodeling - Metabolism (Glycolysis/Lactate Production)	miRNA	miR-378aetc.	Regulate glucose uptake (Correlate with/Target GLUT expression)	Priyadharshini et al. (2023)
	lncRNA	DANCR	Upregulates HK2 (Sponges miR-125b-5p)	Shi et al. (2020)
	lncRNA	TMEM105	Enhances LDHA expression (Sponges miR-1208)	Han et al. (2023)
	lncRNA	SNHG7, LINRIS	Stimulate glycolysis (via c-MYC axis regulation)	Liu et al. (2022c), Jiang et al. (2021b)
TME Remodeling - Myeloid Cells (MDSCs and TAMs)	miRNA	miR-155, miR-21	Enhance MDSC function and expansion (Target SHIP-1, PTEN - > indirectly upregulate STAT3)	Li et al. (2014), Sun et al. (2022b)
	lncRNA	lnc-C/EBPβetc.	Influence MDSC differentiation and suppressive gene expression	Leija et al. (2019)
	lncRNA	RNCR3, Olfr29-ps1	Promote MDSC generation/function (Sponge miR-185-5p, miR-214-3p)	Leija et al. (2019)
	lncRNA	MALAT1	Promote M2 TAM polarization and angiogenesis (Sponges miR-140 - > upregulates VEGF-A)	Hou et al. (2020)
	lncRNA	NEAT1	Promote M2 TAM polarization (Sponges miR-214 - > upregulates B7-H3)	Gao et al. (2020)
	Exosomal	miR-21, TUC339, etc.	Promote M2 TAM polarization/MDSC generation (Tumor-derived exosomes modulate myeloid cells)	Safarzadeh et al. (2020), Mohapatra et al. (2021), Li et al. (2022)
Intrinsic Resistance - Epigenetics	miRNA	miR-148a-3p	Decreases RUNX3 promoter methylation (Targets DNMT1 - LSCC context)	Jili et al. (2016)
	lncRNA	HOTAIR	Silences Tumor Suppressor Genes (Recruits PRC2/EZH2)	Wu et al. (2015)
	lncRNA	IFITM4P	Inhibits PTEN transcription (Enhances KDM5A binding to PTEN promoter)	Shi et al. (2022)
Intrinsic Resistance - CSCs	miRNA	miR-21	Enhances stemness and invasion (Upregulates Oct4, Sox2, Nanog, β-catenin; targets CD44^+^ CSCs)	Jaksic Karisik et al. (2025)
	lncRNA	HOTAIR	Promotes stemness (High expression in OSCC CSCs; regulates stemness markers)	Lu et al. (2017)
	lncRNA	H19	May promote stemness (Potential EZH2 regulation)	Rajendran et al. (2024)
	lncRNA	MALAT1	Promotes EMT and stemness (Via activation of Wnt/β-catenin pathway - TSCC context)	Shao et al. (2022)

### 2.1 ncRNA regulation of immune checkpoint pathways

The PD-1/PD-L1 pathways are critical negative regulators of T cell activation and function, acting as crucial “brakes” on the immune system to maintain self-tolerance (Freeman et al., 2000; Pardoll, 2012; Tivol et al., 1995). PD-L1, often upregulated on tumor cells or immune cells within the TME, binds to PD-1 on activated T cells, suppressing their proliferation, cytokine production, and cytotoxic activity (Pauken and Wherry, 2015; Wherry et al., 2007). While ICIs targeting PD-1/PD-L1 pathways have revolutionized cancer therapy, resistance remains a major challenge (Sharma et al., 2017). Tumor cells commonly evade immune surveillance by exploiting the PD-1/PD-L1 immune checkpoint pathway. Emerging evidence indicates that ncRNAs are key players in regulating the expression and function of these checkpoint molecules, offering potential explanations for resistance and new therapeutic targets (Chen B. et al., 2022).

#### 2.1.1 Specific miRNAs regulating checkpoints in OSCC

**FIGURE 2:**
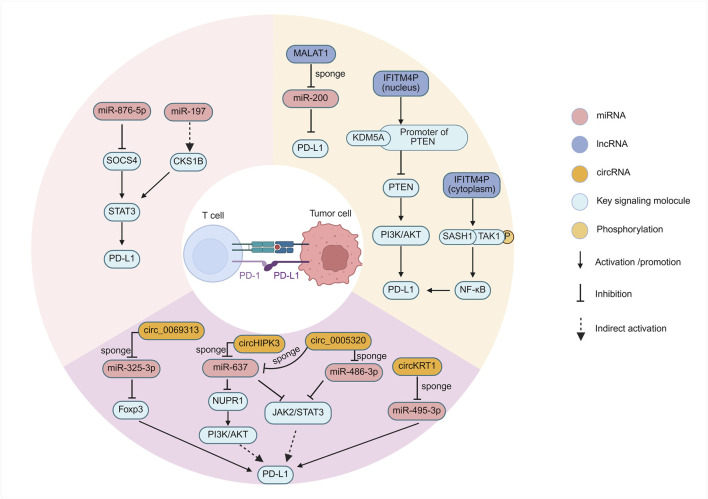
ncRNA-mediated regulation of PD-L1 in OSCC.

miRNAs are potent regulators of immune checkpoint expression (Vaxevanis et al., 2024). Research involving OSCC patient samples has revealed an inverse correlation between the expression levels of miR-197 and PD-L1 protein (Ahn et al., 2017). Although the direct binding of miR-197 to the PD-L1 3′UTR in OSCC cells requires further validation, studies in non-small cell lung cancer (NSCLC) suggest a potential regulatory pathway involving miR-197, CKS1B (CDC28 protein kinase regulatory subunit 1B), and STAT3 (Signal Transducer and Activator of Transcription 3), where miR-197 downregulation leads to increased CKS1B/STAT3 activity and subsequent PD-L1 upregulation (Fujita et al., 2015). Given that STAT3 signaling is relevant in OSCC (Thomas et al., 2024; Jiang et al., 2024), this pathway provides a plausible, albeit indirectly supported, mechanism for miR-197-mediated PD-L1 regulation in OSCC (Figure 2). The implication is that loss of miR-197 expression could contribute to immune evasion by relieving the suppression of PD-L1.

In OSCC, functional assays including dual-luciferase reporter assays have confirmed that miR-495-3p directly targets PD-L1 mRNA, and its downregulation or functional sequestration in OSCC contributes to increased PD-L1 expression (Yang et al., 2021). Crucially, this regulation was shown to be part of a larger ceRNA network involving a circular RNA, CircKRT1, which acts as a sponge for miR-495-3p (Yang et al., 2021). This finding provides a clear example of how miRNA directly mediates the suppression of PD-L1 in OSCC and how this suppression can be counteracted by other ncRNAs.

Furthermore, a study in OSCC identified upregulated miR-876-5p expression, which significantly correlated with tumor recurrence and poor clinical prognosis. Functionally, miR-876-5p was shown to promote OSCC cell proliferation, migration, stemness, and chemoresistance. Mechanistically, miR-876-5p directly targets the tumor suppressor SOCS4 (Suppressor of cytokine signaling 4), leading to sustained activation of the STAT3 pathway and subsequent upregulation of PD-L1, thereby facilitating tumor immune escape (Peng et al., 2025). This study establishes a clear connection between miR-876-5p and various malignant phenotypes as well as immune evasion through the SOCS4/STAT3/PD-L1 axis in OSCC. This underscores the potential for miRNAs to act as critical nodes integrating distinct oncogenic mechanisms, suggesting that targeting miR-876-5p could concurrently counteract multiple malignant characteristics of OSCC.

Several other miRNAs have been implicated in regulating PD-L1 in various cancer types, including miR-138-5p, miR-513, miR-570, miR-34a, and miR-200 (Chen et al., 2016; Deng et al., 2023; Song N. et al., 2020; Zhao et al., 2016; Chen et al., 2014; Kishta et al., 2025). However, their specific roles and direct interactions with PD-L1 mRNA within the OSCC context have not yet been definitively established based on the available data. While miRNAs like miR-21 are frequently upregulated in OSCC and associated with poor prognosis and cancer stem cell properties (Jaksic Karisik et al., 2025), and others like miR-375, miR-92b-3p, and miR-486-5p are downregulated and associated with recurrence (Yan et al., 2017), their direct regulatory impact on PD-1, PD-L1, or CTLA-4 expression in OSCC remains largely uninvestigated.

The available evidence indicates that miRNAs provide a significant layer of post-transcriptional control over immune checkpoint expression, particularly PD-L1, within OSCC cells. The downregulation or sequestration of specific tumor-suppressive miRNAs, such as miR-197 and miR-495-3p, appears to directly contribute to the upregulation of PD-L1 on OSCC cells. This mechanism represents a direct route by which miRNA dysregulation can facilitate immune evasion. Since PD-L1 binding to PD-1 on T cells suppresses anti-tumor immunity (Hirai et al., 2017), the loss of miRNA-mediated inhibition of PD-L1 allows tumor cells to enhance this immunosuppressive signaling.

#### 2.1.2 lncRNA networks regulating checkpoints in OSCC

In the context of various cancers, lncRNAs have been demonstrated to participate in the regulation of immune checkpoint pathways, providing a framework for potential mechanisms in OSCC. For example, multiple lncRNAs have been shown to modulate PD-L1 expression by acting as microRNA sponges. In pancreatic cancer, LINC00473 promotes PD-L1 expression by sponging miR-195-5p (Zhou et al., 2019), while in hepatocellular carcinoma, PCED1B-AS1 upregulates PD-L1 via miR-194-5p (Fan et al., 2021). Similarly, the lncRNA KCNQ1OT1 regulates PD-L1 by sponging miR-15a in prostate cancer and miR-506 in hepatocellular carcinoma (Chen et al., 2020; Zhang et al., 2020), and it also affects the miR-30a/USP22 axis in colorectal cancer, which has implications in cancer progression though direct regulation of PD-L1 protein stability via this specific axis requires further clarification (Xian et al., 2021). Beyond these mechanisms, Some lncRNAs indirectly regulate checkpoints by influencing signaling pathways. CASC11 affects PD-L1 by activating NF-κB and PI3K/AKT/mTOR signaling through the EIF4A3/E2F1 pathway in hepatocellular carcinoma (Song H. et al., 2020), while HOTTIP induces IL-6 production, activating the IL-6/JAK/STAT3/PD-L1 pathway in neutrophils within the ovarian cancer microenvironment (Shang et al., 2019). Furthermore, lncRNAs employ epigenetic mechanisms, such as HOTAIR potentially participating in PRC2-mediated silencing (Balas et al., 2021), and LncMX1–215 inhibiting PD-L1 and Galectin-9 expression by interfering with GCN5-mediated H3K27 acetylation in head and neck squamous cell carcinoma (Ma et al., 2020). Even more directly, lncRNAs like Lnc-Tim3 can bind to immune checkpoint proteins (e.g., TIM-3), interfere with their downstream molecular interactions, and consequently affect T cell apoptosis in hepatocellular carcinoma (Ji et al., 2018). These examples from other cancers highlight plausible, yet unconfirmed, regulatory axes that warrant investigation within the OSCC-specific context.

In the field of OSCC, research investigating the lncRNA-mediated regulation of immune checkpoints has also yielded notable advances (Figure 2). During the progression from oral leukoplakia, a premalignant lesion, to OSCC, the long non-coding RNA IFITM4P was found to be significantly upregulated and promotes PD-L1 expression through a unique dual mechanism dependent on its subcellular localization (Shi et al., 2022). In the cytoplasm, IFITM4P functions as a molecular scaffold, facilitating the interaction between SASH1 and TAK1, leading to TAK1 phosphorylation at Thr187. This subsequently activates the NF-κB signaling pathway, evidenced by p65 phosphorylation at Ser536, which in turn induces PD-L1 gene transcription. By contrast, within the nucleus, IFITM4P enhances the binding of the histone demethylase KDM5A to the promoter of the tumor suppressor gene PTEN, thereby inhibiting PTEN transcription (Shi et al., 2022). The resulting loss or inactivation of PTEN function is known to activate the PI3K/AKT pathway, which subsequently leads to the indirect upregulation of PD-L1 expression. This discovery not only reveals the pro-oncogenic role of IFITM4P in oral carcinogenesis but also elegantly demonstrates how the same lncRNA can utilize distinct molecular mechanisms—signal transduction modulation in the cytoplasm and epigenetic regulation in the nucleus—to synergistically upregulate the key immune checkpoint molecule PD-L1. The complexity and precision of this regulatory mechanism highlight the central role of lncRNAs in cellular regulatory networks. Intriguingly, in mouse models, tumors exhibiting high IFITM4P expression demonstrated enhanced sensitivity to anti-PD-1 antibody therapy (Shi et al., 2022), suggesting that IFITM4P could serve as a potential biomarker for predicting ICI efficacy, despite its function in promoting PD-L1 expression.

LncRNA MALAT1, a well-documented oncogenic driver upregulated in OSCC and other malignancies, promotes tumor progression through multifaceted mechanisms including PD-L1 mediated immune evasion (Chang and Hu, 2018; Xiao et al., 2020). Its overexpression correlates strongly with advanced disease stages, metastatic propensity, and dismal clinical outcomes (Fu et al., 2020). Mechanistically, its regulation of PD-L1 is complex, with several potential pathways elucidated in other cancers that may also be relevant to OSCC: (I) ceRNA activity by sponging miR-200 family members that normally suppress PD-L1 translation, MALAT1 derepresses PD-L1 production, enabling cancer cells to evade T cell surveillance in lung cancer (Xu et al., 2024); (II) MALAT1 interacts with epigenetic regulatory complexes. Specifically, its association with EZH2, the catalytic subunit of the PRC2 complex, has been demonstrated in cancers such as pancreatic cancer (Cheng et al., 2018) and castration-resistant prostate cancer (Wang et al., 2015). This interaction suggests that MALAT1 might influence PD-L1 expression by guiding epigenetic modifications or by serving as a regulatory protein scaffold, although the precise mechanism in OSCC requires further investigation; (III) At the epitranscriptomic level, the N6-methyladenosine (m6A) writer protein METTL3 has been shown in pancreatic cancer to enhance MALAT1 stability. This modification indirectly increases PD-L1 levels by increasing the abundance of the MALAT1 transcript (Song et al., 2022). Although direct evidence for some of these pathways in OSCC is still emerging, these convergent mechanisms, validated across different cancers, collectively suggest that MALAT1 is a potential regulator of immunosuppressive signaling in OSCC.

HOX transcript antisense intergenic RNA (HOTAIR) is another lncRNA frequently overexpressed in numerous cancers, including OSCC, where its high levels are associated with advanced stage, metastasis, poor prognosis, and the promotion of epithelial-mesenchymal transition (EMT) (Chen et al., 2021). However, the mechanisms by which HOTAIR upregulates PD-L1 have been elucidated primarily in other tumor types, and their applicability to OSCC requires specific investigation. HOTAIR employs several mechanisms to upregulate PD-L1 expression and promote immune evasion in cancer. One prominent mechanism involves acting as a ceRNA or microRNA sponge. In laryngeal squamous cell carcinoma, HOTAIR sponges hsa-miR-30a-5p, thereby relieving the inhibition of its target GRP78 (HSPA5), which subsequently leads to increased PD-L1 protein levels (Yuan et al., 2022). Although not yet confirmed in OSCC, this suggests a plausible regulatory axis. Another key mechanism is the activation of signaling pathways that control PD-L1 transcription. In glioma, HOTAIR has been shown to activate the NF-κB pathway, potentially by suppressing upstream inhibitors like UBXN1, leading to increased NF-κB nuclear translocation and subsequent binding to the PD-L1 promoter to drive its expression (Wang et al., 2021). Furthermore, HOTAIR can be transferred between cells via extracellular vesicles (EVs), as observed in esophageal cancer, where EV-delivered HOTAIR modulates the TME and upregulates PD-L1 in recipient cells, contributing to chemoresistance and immune escape (Tuersong et al., 2025). While HOTAIR is well-known for recruiting the PRC2 complex for epigenetic silencing, its direct regulation of PD-L1 appears to rely more on these post-transcriptional and signaling-based mechanisms found in other cancers, highlighting the need for studies to validate these pathways specifically within the OSCC context.

The involvement of lncRNAs adds significant complexity to the regulation of immune checkpoints in OSCC. They function as crucial integrators, connecting diverse upstream cellular signals—ranging from inflammatory responses triggered by the microenvironment (e.g., LPS/TLR4 activating IFITM4P (Ji et al., 2024)) to aberrant activation of developmental pathways like Wnt/β-catenin driving EMT (Shao et al., 2022)—to downstream effectors, including potentially the expression levels of immune checkpoint molecules like PD-L1. This positions lncRNAs as central mediators translating the cellular state and environmental cues into specific immune evasion phenotypes.

#### 2.1.3 CircRNAs regulating checkpoints in OSCC

Specific circRNAs have been identified that directly regulate the expression of PD-L1 in OSCC, consequently contributing to tumor immune evasion (Figure 2). CircKRT1 is significantly upregulated in OSCC tissues and cell lines, such as Cal-27, SCC-25, SCC-9, and HSC-3 (78). Functional studies have demonstrated that circKRT1 acts as a sponge for miR-495-3p, sequestering this miRNA and thus relieving its inhibitory effect on the target PD-L1 mRNA, which leads to the upregulation of PD-L1 protein expression. Elevated PD-L1 levels subsequently inhibit the anti-tumor activity of CD8^+^ T cells, promote OSCC cell proliferation, migration, invasion, and the EMT process, ultimately facilitating immune escape and accelerating tumor progression. Clinical data also indicate that high expression of circKRT1 is closely associated with poor prognosis in OSCC patients (Yang et al., 2021).

Another extensively studied circRNA, hsa_circ_0069313 (also designated as circ_0069313), is also highly expressed in OSCC tissues and cells, and its expression level is significantly associated with advanced clinical stage and poor prognosis (Chen Y. et al., 2022). The mechanism of this circRNA involves a dual role in promoting immune evasion. First, hsa_circ_0069313 competitively binds to miR-325-3p, thereby preventing the miRNA-mediated degradation of Forkhead box P3 (Foxp3) mRNA, resulting in increased Foxp3 protein levels within OSCC cells themselves (Chen Y. et al., 2022). Increased Foxp3 subsequently upregulates the expression of the immune checkpoint protein PD-L1 on the surface of cancer cells (Mulder et al., 2025; Xu et al., 2023). This completes a key immunosuppressive cascade: the elevated PD-L1 on tumor cells engages with its receptor, PD-1, on activated cytotoxic CD8^+^ T cells. This interaction recruits the phosphatase SHP-2 to the PD-1 cytoplasmic domain, which in turn dampens the T-cell receptor (TCR) signaling cascade. The ultimate consequence is the induction of T-cell exhaustion, a state of dysfunction characterized by reduced proliferation, impaired cytokine production (e.g., IFN-γ, TNF-α), and diminished cytotoxic capacity, thereby allowing the tumor to escape immune-mediated destruction (Celis-Gutierrez et al., 2019). This is consistent with clinical findings where high hsa_circ_0069313 levels negatively correlate with CD8^+^ T cell infiltration scores in OSCC tissues (Chen Y. et al., 2022). Second, hsa_circ_0069313 can also be packaged into exosomes secreted by OSCC cells and subsequently delivered to Tregs within TME. Inside Tregs, exosome-derived hsa_circ_0069313 continues to function as a sponge for miR-325-3p, stabilizing Foxp3 expression (Chen Y. et al., 2022). This stabilization is critical, as foundational studies have established Foxp3 as the master regulatory gene that programs the entire immunosuppressive lineage and function of Tregs (Fontenot et al., 2003; Hori et al., 2003). This fundamental program, driven by sustained Foxp3 expression, includes the upregulation of characteristic molecules like CD25, GITR, and the inhibitory receptor CTLA-4 (Fontenot et al., 2003). Functionally, it renders Tregs hyporesponsive to TCR stimulation while empowering them to actively suppress the proliferation of other effector T cells through mechanisms requiring cell-to-cell contact (Fontenot et al., 2003; Hori et al., 2003). By reinforcing this core suppressive program in Tregs within the tumor microenvironment, the exosome-delivered circRNA enhances their capacity to inhibit anti-tumor effector cells. This dual action—enhancing PD-L1 on tumor cells and reinforcing the suppressive machinery of Tregs—establishes hsa_circ_0069313 as a critical driver of immunosuppression in OSCC. In addition to the circRNAs mentioned above that directly regulate PD-L1, some circRNAs dysregulated in OSCC, while not yet directly proven to regulate PD-L1, are implicated in modulating key signaling pathways, such as the PI3K/AKT and JAK/STAT pathways, which are known to be closely involved in PD-L1 regulation (Sanchez et al., 2024; Wu et al., 2024). These suggest that circRNAs might indirectly influence the immune checkpoint status in OSCC. CircHIPK3, which is highly expressed in OSCC, reportedly activates the PI3K/AKT pathway via the miR-637/NUPR1 axis, a critical pathway for PD-L1 expression regulation (Jiang W. et al., 2021). Similarly, the highly expressed circ_0005320 can activate the JAK2/STAT3 pathway by sponging miR-486-3p and miR-637; STAT3 signaling is also a key inducer of PD-L1 transcription which is mentioned above (Zheng et al., 2022). Nevertheless, these potential indirect regulatory links still require more direct experimental evidence for confirmation.

Collectively, based on the available research data, the study of direct regulation of immune checkpoints, particularly PD-L1, by circRNAs in OSCC is still in its relatively early stages. The number of clearly reported and thoroughly investigated examples is limited, and the observed mechanisms predominantly involve the miRNA sponge effect. This current situation may reflect the maturity of this research field, while also suggesting that the miRNA sponge mechanism could be a primary mode of circRNA-mediated PD-L1 regulation in OSCC.

### 2.2 T Cell exhaustion and functional suppression

#### 2.2.1 CD8^+^ T Cell exhaustion

Cytotoxic CD8^+^ T lymphocytes (CTLs) are critical for eliminating cancer cells (Zabeti and Vahidi, 2024). However, within the TME, chronic antigen exposure and immunosuppressive signals can drive CTLs into a state of exhaustion (Chen et al., 2025; Zarour, 2016). Persistent antigen stimulation leads to sustained high expression of PD-1 on the T cell surface (Freeman et al., 2006), while the interaction between PD-1 and PD-L1, represents a critical pathway for inducing T cell exhaustion (Sasaki et al., 2024). As previously mentioned, miR-495-3p directly targets the PD-L1 mRNA. The downregulation or trapping of this miRNA in OSCC results in increased expression of PD-L1 (Yang et al., 2021). In non-small cell lung cancer, circRNA-002178 acts as a ceRNA sponge for specific miRNAs, subsequently upregulating the expression of both PD-1 and PD-L1 (Li Z. et al., 2024). Furthermore, in nasopharyngeal carcinoma, circBART2.2 upregulates PD-L1 expression and induces T cell apoptosis (Li Z. et al., 2024). These findings indicate that ncRNAs are involved in CTL-mediated immunosuppression associated with PD-1/PD-L1 interaction.

T cell immunoglobulin and mucin-domain containing-3 (TIM-3) is another critical marker of T cell exhaustion, often co-expressed with PD-1 on the most functionally defective tumor-infiltrating lymphocytes (TILs) (Sakuishi et al., 2010). In OSCC, the expression level of the lncRNA CRNDE is significantly elevated within tumor-infiltrating CD8^+^ T cells and shows a negative correlation with interferon-gamma (IFN-γ) production (Ai SW et al., 2024). Further mechanistic investigation revealed that CRNDE functions as a ceRNA by competitively binding to miR-545-5p, which relieves the suppressive effect of miR-545-5p on TIM-3 (encoded by the HAVCR2 gene) mRNA. This results in upregulated TIM-3 protein expression, which consequently promotes CD8^+^ T cell exhaustion and diminishes their anti-tumor cytotoxic capabilities (Ai et al., 2021). This CRNDE/miR-545-5p/TIM-3 axis provides concrete molecular evidence for lncRNA-mediated regulation of T cell exhaustion in OSCC through ceRNA network interactions. Additionally, in hepatocellular carcinoma, the lncRNA Lnc-Tim3 was found to be highly expressed in exhausted CD8^+^ T cells. It can directly bind to the TIM-3 receptor, preventing the interaction between TIM-3 and Bat3, and inhibiting the downstream Lck/NFAT1/AP-1 signaling pathway. Concurrently, it promotes Bat3 nuclear translocation, enhancing the p300-dependent transcriptional activation of anti-apoptotic genes (such as MDM2 and Bcl-2) by p53 and RelA, thereby exacerbating the state of T cell exhaustion (Ji et al., 2018). Therefore, modulating miRNAs targeting TIM-3 or associated lncRNAs holds therapeutic potential for reversing T cell exhaustion (Zabeti and Vahidi, 2024).

Lymphocyte-activation gene 3 (LAG-3) is an inhibitory receptor expressed on the surface of various lymphocytes; it negatively regulates T cell activation and effector function through binding to Major Histocompatibility Complex class II (MHC-II) molecules (Wuerdemann et al., 2020). LAG-3 is frequently co-expressed with PD-1 on exhausted T cells and synergistically drives their state of exhaustion (Sakuishi et al., 2010). Studies have shown that the combined action of LAG-3 and PD-1 can drive the expression of the exhaustion-associated transcription factor TOX. Furthermore, effective enhancement of anti-tumor immunity upon blockade of both LAG-3 and PD-1 requires autocrine interferon-gamma (IFN-γ) signaling (Andrews et al., 2024). Clinical trials have also confirmed that combined blockade of PD-1 and LAG-3 (e.g., nivolumab plus relatlimab) demonstrates superior efficacy compared to monotherapy blockade in tumors such as melanoma (Andrews et al., 2024). In oropharyngeal squamous cell carcinoma (OPSCC), LAG-3 expression on TILs is significantly associated with CD8^+^ T cell infiltration, particularly in HPV-positive cases (Wuerdemann et al., 2020). Although specific reports on the direct regulation of LAG-3 expression by ncRNAs in OSCC are currently lacking, considering the synergistic action of LAG-3 and PD-1, ncRNAs that regulate either pathway individually or both simultaneously are likely to significantly impact the state of T cell exhaustion.

In other disease models, miRNAs such as miR-31 and miR-155 have been demonstrated to be involved in the regulation of pathways associated with T cell exhaustion (Li and Wang, 2022). CircRNAs also play significant roles. Exosome-derived circUSP7 induces CD8^+^ T cell dysfunction and resistance to anti-PD-1 therapy through the regulation of the miR-934/SHP2 axis. Moreover, the expression and function of circRNAs themselves can potentially be modulated by RNA modifications, such as m6A, which may in turn affect T cell activation and proliferation (Li Z. et al., 2024).

#### 2.2.2 Treg expansion and immunosuppression

In OSCC, increased infiltration of Treg cells is generally associated with poor prognosis, and they are key contributors to the formation of an immunosuppressive microenvironment and the development of immunotherapy resistance (Mohd Faizal et al., 2025). Treg cells, typically characterized by the expression of CD4, CD25, and the transcription factor FOXP3, represent one of the major immunosuppressive cell populations within TME (Ghods et al., 2021). Multiple ncRNAs have been found to directly or indirectly regulate the expression of FOXP3. MiR-125b inhibits FOXP3 expression by targeting the 3′ untranslated region (3′ UTR) of its mRNA (Wang et al., 2018). Conversely, miR-4281 can bind to the human FOXP3 promoter region and upregulate its protein expression (Zhang et al., 2018). In gastric cancer, both the MKL1/miR-34a/FOXP3 axis and miR-133a-3p suppress FOXP3 expression (Wang et al., 2024). In esophageal squamous cell carcinoma (ESCC), lncRNA MEG3 indirectly upregulates FOXP3 expression by inhibiting the ubiquitination of p53, an upstream regulator of miR-149-3p, thereby enhancing the immunosuppressive activity of Tregs (Xu et al., 2021). In hepatocellular carcinoma, lnc-EGFR promotes immune evasion by stabilizing the EGFR protein, which enhances the downstream AP-1/NF-AT1 signaling pathway and drives Treg differentiation (Jiang et al., 2017). In OSCC, circRNA has_circ_0069313 has been found to promote tumor immune evasion through the miR-325-3p/Foxp3 axis, a mechanism impacting both OSCC cells and Tregs (Chen Y. et al., 2022). This multi-layered and intricate regulatory network underscores the crucial role of ncRNAs in maintaining the immunosuppressive state within TME.

Specific ncRNAs have been directly linked to Treg-mediated immunotherapy resistance. CircQSOX1-mediated Treg induction contributes to resistance against anti-CTLA-4 therapy (Liu Z. et al., 2022), whereas circ_0069313 promotes immune evasion in OSCC by influencing Tregs (Chen Y. et al., 2022). This suggests that assessing the expression levels of specific ncRNAs within Tregs could aid in predicting patient responses to certain immune checkpoint inhibitors. Furthermore, targeting these pivotal ncRNA molecules may represent a novel strategy to overcome Treg-mediated immunosuppression and therapeutic resistance.

### 2.3 Remodeling of the immunosuppressive microenvironment

#### 2.3.1 Metabolic reprogramming

Metabolic reprogramming is one of the ten hallmarks of cancer (Meng et al., 2024). To meet the energy and biosynthetic demands associated with rapid proliferation, tumor cells exhibit significant metabolic shifts. Most notably, they preferentially engage in glycolysis even under aerobic conditions, resulting in substantial lactate accumulation (Mao et al., 2024). This microenvironment is typically characterized by high lactate levels, low pH, hypoxia, and competition for nutrients. These factors collectively suppress the function of effector T cells while promoting the activity of inhibitory cells, including Tregs and myeloid-derived suppressor cells (MDSCs), consequently leading to immunosuppression and resistance to immunotherapy (Zhang X. et al., 2024). As key regulators of gene expression, ncRNAs play pivotal roles in driving and sustaining tumor metabolic reprogramming. In OSCC, specific ncRNAs have been demonstrated to directly engage with and remodel glycolysis by targeting key metabolic molecules. The m6A-modified circFOXK2 has been shown to enhance the stability of glucose transporter 1 (GLUT1) mRNA, thereby promoting carcinogenesis in OSCC (Cui Y. et al., 2023). Another study identified that the upregulated circMDM2 in OSCC promotes cell proliferation and glycolysis by modulating the miR-532-3p/HK2 axis (Zheng et al., 2020). These findings directly link specific circRNAs to core aspects of glycolysis in OSCC.

Multiple miRNAs have been found to regulate the expression of glucose transporters (GLUTs), consequently influencing glucose uptake and the glycolytic rate in OSCC. For instance, miR-378a, miR-182-5p, miR-340, miR-150-5p, and miR-200c have been reported to correlate with GLUT1 expression, whereas miR-23a-3p is associated with GLUT3 (Priyadharshini et al., 2023). Dysregulation of these miRNAs leads to aberrant GLUT expression, subsequently impacting the glycolytic activity of OSCC cells and tumor progression. Moreover, lncRNAs can regulate key enzymes within the glycolytic pathway—such as hexokinase 2 (HK2), lactate dehydrogenase A (LDHA), and pyruvate kinase M2 (PKM2)—through diverse mechanisms. LncRNA HITT inhibits the oligomerization of PKM2, thereby suppressing lactate production (Zhao et al., 2022); lncRNA DANCR upregulates HK2 by sponging miR-125b-5p (Shi et al., 2020); and lncRNA TMEM105 enhances LDHA expression by acting as a sponge for miR-1208 (Han et al., 2023). These regulatory actions directly impact glycolytic flux and lactate generation.

ncRNAs can regulate key metabolic transcription factors, such as hypoxia-inducible factor-1α (HIF-1α) and c-Myc, both of which are crucial drivers of the Warburg effect (Alamoudi, 2025). LncRNA SNHG7 stimulates glycolysis via the SRSF1/c-MYC axis (Liu J. et al., 2022), while lncRNA LINRIS maintains glycolysis by stabilizing the IGF2BP2 protein, leading to upregulated c-Myc expression (Jiang T. et al., 2021). Furthermore, ncRNAs can influence key signaling pathways governing cellular metabolism, including the AMPK, mTOR, and Hippo/YAP pathways. LncRNA MACC1-AS1 influences glycolysis by activating the AMPK pathway (Zhao Y. et al., 2018), while knockdown of lncRNA MALAT1 suppresses phosphorylation within the mTOR pathway, thereby inhibiting the Warburg effect (Malakar et al., 2019).

Through these multifaceted regulatory mechanisms, ncRNAs drive metabolic reprogramming in OSCC, characterized by elevated glycolysis and lactate accumulation, shaping an immunosuppressive TME, which directly suppresses the activity of anti-tumor immune cells, particularly T cells, inducing their dysfunction or exhaustion, thereby establishing a key foundation for immunotherapy resistance.

#### 2.3.2 Myeloid cell-mediated barrier

Myeloid cells, particularly MDSCs and tumor-associated macrophages (TAMs), represent major immunosuppressive populations within TME (Mo et al., 2023). Through diverse mechanisms, myeloid cells suppress T cell responses, promote tumor progression and immune evasion, thereby constituting a formidable barrier to effective immunotherapy (Huang et al., 2025). ncRNAs play crucial roles in regulating the differentiation, recruitment, polarization, and function of these myeloid cells.

MDSCs constitute a heterogeneous population of immature myeloid cells characterized by potent immunosuppressive capabilities (Sun Y. et al., 2022). Within the TME of OSCC, MDSCs suppress T cell proliferation and function through the production of factors such as arginase 1 (ARG1), inducible nitric oxide synthase (iNOS), and reactive oxygen species (ROS), while also inducing Treg generation (Li C. et al., 2024). ncRNAs regulate the generation, recruitment, and function of MDSCs through intricate networks. miR-17-5p and miR-20a, by targeting STAT3, can downregulate the suppressive functions of MDSCs, including ROS production (Liang et al., 2022). Similarly, miR-6991-3p targets STAT3, thereby inhibiting MDSC expansion (Sun et al., 2019). Conversely, miR-155 and miR-21 enhance MDSC function and expansion by indirectly upregulating STAT3, achieved through targeting SHIP-1 (Li et al., 2014) and PTEN (Sun Q. et al., 2022), respectively. miR-449c influences the expansion of monocytic MDSCs (M-MDSCs) through the regulation of STAT6 (Han et al., 2020). LncRNAs such as lnc-C/EBPβ, lnc-CHOP, and RUNXOR influence MDSC differentiation and the expression of suppressive genes (Arg-1, iNOS, NOX2) through direct binding to, or indirect regulation of, key transcription factors including C/EBPβ, CHOP, and RUNX1 (Leija et al., 2019). Furthermore, lncRNAs such as RNCR3 and Olfr29-ps1 function by sponging specific miRNAs (miR-185-5p and miR-214-3p), thereby relieving the inhibitory effects of these miRNAs on downstream pro-MDSC generation/function genes, including CHOP and MyD88 (Leija et al., 2019). Moreover, evidence has also demonstrated that tumor cell-derived exosomes can transfer ncRNAs to monocytes, promoting their differentiation into MDSC-like cells and augmenting their immunosuppressive capabilities (Safarzadeh et al., 2020).

As one of the most abundant immune cell populations in the TME, TAMs typically exhibit an M2-like polarization state. They possess functions including the promotion of tumor angiogenesis, matrix remodeling, immunosuppression, and tumor metastasis (Peltanova et al., 2019). The polarization state of macrophages (pro-inflammatory M1 versus anti-inflammatory/pro-tumoral M2) is governed by diverse signals within the TME, with ncRNAs acting as critical regulators in this process (Zhou et al., 2022). MiRNAs such as miR-155 and miR-142-3p typically promote M1 polarization, exerting anti-tumor effects; conversely, miR-21, miR-29a-3p, miR-145, and miR-301a-3p tend to drive M2 polarization, contributing to pro-tumor functions (Mohapatra et al., 2021). LncRNAs also play significant roles: lncRNA MALAT1 promotes M2 polarization and angiogenesis by sponging miR-140, leading to the upregulation of VEGF-A (Hou et al., 2020). Similarly, lncRNA NEAT1 induces M2 polarization by sponging miR-214, resulting in increased B7-H3 expression (Gao et al., 2020). Tumor cells can also deliver specific miRNAs (e.g., miR-21, miR-29a-3p) or lncRNAs (e.g., TUC339, RPPH1) to macrophages via exosomes, thereby promoting their polarization towards an M2 phenotype (Mohapatra et al., 2021). Reciprocally, TAMs can transfer ncRNAs back to tumor cells via exosomes, influencing tumor cell proliferation, invasion, and drug resistance (Li et al., 2022). This bidirectional communication via exosomal ncRNAs constitutes a crucial mechanism underlying the co-evolution of tumor and immune cells within the TME.

In summary, ncRNAs exert fine-tuned control over the generation, recruitment, and functional status of two critical myeloid suppressor cell populations—MDSCs and TAMs—by modulating key transcription factors, signaling pathways, and acting as miRNA sponges. Notably, the exosome-mediated transfer of ncRNAs represents a significant pathway through which tumor cells actively sculpt the immunosuppressive microenvironment. This myeloid cell barrier, meticulously orchestrated by ncRNAs, effectively suppresses anti-tumor T cell responses and represents a major cause of immunotherapy resistance in OSCC.

### 2.4 Intrinsic resistance mechanisms in tumor cells

In addition to mediating resistance through effects on immune cells and TME, ncRNAs can also directly regulate the intrinsic biological characteristics of tumor cells, thereby conferring inherent resistance to immunotherapy. These mechanisms primarily involve epigenetic remodeling and the maintenance of cancer stem cell (CSC) characteristics.

#### 2.4.1 Epigenetic remodeling

In contrast to genetic mutations that directly change nucleotide sequences, epigenetic modifications—encompassing DNA methylation, histone modifications, and ncRNA-mediated regulation—modulate gene expression patterns without modifying the DNA sequence. These modifications represent one of the key mechanisms underlying tumorigenesis, progression, and the development of therapeutic resistance (Bais, 2019). Aberrant epigenetic patterns are prevalent in OSCC, exemplified by the hypermethylation of promoter regions in tumor suppressor genes (TSGs), leading to their silencing, or by abnormal histone modifications that result in oncogene activation (Kim et al., 2019; Adimulam et al., 2021). There is intricate crosstalk between ncRNAs and epigenetic modifications, which collectively shape the resistant phenotype of tumor cells (Huang and Aghaei-Zarch, 2024).

Certain ncRNAs, especially miRNAs, can directly target and regulate the expression of key epigenetic modifying enzymes, including DNA methyltransferases (DNMTs), histone deacetylases (HDACs), histone methyltransferases (HMTs), and lysine demethylases (KDMs) (Bure et al., 2022; Gazdzicka et al., 2020). In laryngeal squamous cell carcinoma, miR-148a-3p targets DNMT1, thereby decreasing the methylation level of the tumor suppressor gene RUNX3 (Jili et al., 2016). Furthermore, acting as scaffolds or guides in epigenetic regulation, lncRNAs can associate with chromatin-modifying complexes, such as the Polycomb Repressive Complex 2 (PRC2) containing EZH2, or with other epigenetic enzymes like DNMTs, HDACs, Histone acetyltransferases, and KDMs. By recruiting these complexes/enzymes to specific genomic loci, lncRNAs mediate local histone modifications or DNA methylation, consequently regulating target gene transcription (Lei et al., 2021). The lncRNA HOTAIR, implicated in various cancers including OSCC, suppresses the expression of downstream tumor suppressor genes by recruiting EZH2 and mediating H3K27 trimethylation (Wu et al., 2015). In OSCC, the lncRNA IFITM4P inhibits PTEN transcription by enhancing the binding of KDM5A to the PTEN promoter (Shi et al., 2022).

Conversely, the expression of numerous ncRNA genes is regulated by epigenetic mechanisms, including DNA methylation or histone modifications. In OSCC, the promoter regions of tumor-suppressive miRNAs like miR-137 and miR-193a are frequently silenced via hypermethylation (Kozaki et al., 2008). In contrast, the lncRNA H19 exhibits hypomethylation and elevated expression in OSCC (Rajendran et al., 2024). This bidirectional interplay between ncRNAs and epigenetic machinery establishes a complex regulatory network. Targeting these pivotal ncRNAs or related epigenetic pathways could potentially reverse adverse epigenetic states, reinstate the expression of immune-associated genes, and consequently improve sensitivity to immunotherapy.

#### 2.4.2 Cancer stem cell properties

CSCs represent a small subpopulation within tumors characterized by self-renewal capacity and multipotent differentiation potential. These cells are believed to be the driving force behind tumor initiation, progression, metastasis, recurrence, and resistance to various therapies, including chemotherapy, radiotherapy, and immunotherapy (Chen and Wang, 2019). In OSCC, distinct CSC populations have been identified based on the expression of specific markers, including CD44, ALDH1, CD133, and Bmi-1 (Siqueira et al., 2023). ncRNAs play significant roles in regulating key characteristics of CSCs, and a growing body of research in OSCC has identified specific ncRNA molecules that directly govern the CSC phenotype.

Specific examples of ncRNA regulation in OSCC CSCs include the let-7 family, which inhibits CSC characteristics by targeting factors like Lin28B, ARID3B, and HMGA2, leading to the negative regulation of Oct4, Sox2, and Nanog expression (Hsieh et al., 2020). The miR-200 family inhibits CSC traits by targeting the EMT-associated transcription factors ZEB1/ZEB2, leading to the indirect suppression of the stemness factor Bmi-1 (Hsieh et al., 2020). Conversely, miR-21 enhances stemness maintenance and invasion in CD44^+^ OSCC CSCs by upregulating the expression of Oct4, Sox2, Nanog, and β-catenin (Jaksic Karisik et al., 2025). The lncRNA HOTAIR is highly expressed in OSCC CSCs, and its knockdown inhibits sphere formation ability, reduces ALDH1 activity, and downregulates the expression of stemness markers (Lu et al., 2017). LncRNA H19 might also contribute to stemness maintenance, potentially through mechanisms involving the regulation of EZH2 in OSCC(150). Furthermore, lncRNA MALAT1 promotes EMT and stemness-associated characteristics in tongue squamous cell carcinoma via activation of the Wnt/β-catenin pathway (Shao et al., 2022). ncRNAs can also impact the therapeutic resistance of CSCs by regulating components like ATP-binding cassette (ABC) transporters, anti-apoptotic proteins (such as Bcl-2), and DNA repair pathways. For example, miR-21 suppresses apoptosis in CD44^+^ OSCC CSCs by modulating the Bax/Bcl-2 ratio (Jaksic Karisik et al., 2025). These examples clearly illustrate the central role of lncRNAs within the OSCC CSC regulatory network.

CSCs can evade immune recognition through the downregulation of Major Histocompatibility Complex (MHC)/Human Leukocyte Antigen (HLA) molecules (Zhang et al., 2023). With respect to the regulation of CSC immune evasion, while specific ncRNAs directly controlling MHC expression in CSCs may not be explicitly detailed in the available literature, their extensive regulatory influence over stemness factors (e.g., Sox2, Oct4) and epigenetic mechanisms (which potentially affect MHC expression) suggests their involvement in CSC immune escape pathways. For instance, stemness-maintaining factors like miR-21 or HOTAIR could indirectly contribute to the downregulation of MHC molecules.

Thus, ncRNAs are crucial regulators involved in maintaining the OSCC CSC population and upholding its malignant features. Targeting these critical ncRNAs within CSCs holds the potential not only to directly diminish CSC stemness and therapeutic resistance but also to restore their immunogenicity, rendering them more susceptible to immune system recognition and clearance. Consequently, this approach presents a novel therapeutic strategy for overcoming immunotherapy resistance and preventing tumor recurrence.

## 3 Therapeutic strategies targeting ncRNAs for OSCC immunotherapy

The growing understanding of ncRNAs as critical drivers of immune evasion and therapy resistance in OSCC has spurred interest in developing therapeutic strategies that directly target these molecules (Hashemi et al., 2024). These approaches aim to inhibit the function of pro-tumorigenic ncRNAs or restore the activity of anti-tumorigenic ncRNAs, in order to endogenous anti-tumor immunity or overcoming resistance to existing treatments like ICIs. The summary of therapeutic strategies targeting ncRNAs is listed in Table 2.

**TABLE 2:** Summary of Therapeutic Strategies Targeting ncRNAs for OSCC Immunotherapy.

Therapeutic approach Category	Specific strategy	Mechanism of action	Target examples (ncRNA/Pathway)
Inhibition of Pro-tumorigenic ncRNAs	Antisense Oligonucleotides (ASOs)	Bind to target RNA, leading to its degradation or functional blockade	lncRNAs (MALAT1, HOTAIR), circRNAs (e.g., circKRT1)
	Small Molecule Inhibitors	Target proteins essential for ncRNA biogenesis, processing, or function (indirect inhibition)	EZH2 (affecting HOTAIR function), m6A reader proteins (e.g., YTHDF2)
	CRISPR/Cas9-based Silencing	Utilize dCas9 fused to repressor domains, guided by gRNAs to promoter regions, achieving epigenetic silencing	lncRNA gene promoters (e.g., MALAT1, HOTAIR)
Activation of Anti-tumorigenic ncRNAs	miRNA Mimics	Synthetic double-stranded RNA mimicking endogenous mature miRNA function, silencing target mRNAs via RISC	miR-138-5p (targeting PD-1/CTLA-4), miR-200c (targeting PD-L1/EMT), miR-34a (inhibiting proliferation/CSCs)
	Gene Therapy Vectors	Utilize viral or non-viral vectors to express specific tumor-suppressive ncRNAs	lncRNAs (GAS5, MEG3), miRNA precursors
	Engineered Exosomes/Nanoparticles	Utilize natural or synthetic carriers to deliver therapeutic ncRNAs	lncRNAs (GAS5 in exosomes), miRNA mimics/ASOs in LNPs
Combination Therapies	ncRNA Modulators + ICIs	ncRNAs regulate immune checkpoint expression, TME, and other ICI resistance mechanisms	Inhibiting MALAT1 + anti-PD-1, miR-138 mimics + anti-PD-1/CTLA-4
	Combination of Multiple ncRNA-Targeting Therapies	Simultaneously target ncRNAs involved in different pathways or mechanisms for broader anti-cancer effects	Inhibiting HOTAIR (proliferation) + miR-34a mimics (apoptosis/immunity)
	ncRNA Modulators + Conventional Therapies	Target ncRNAs mediating chemo/radiotherapy resistance	Inhibiting XIST or H19 (chemoresistance) + cisplatin

### 3.1 Inhibitory approaches for pro-tumorigenic ncRNAs

Many ncRNAs, particularly certain lncRNAs and miRNAs (oncomiRs), are upregulated in OSCC and promote tumor growth, metastasis, and immune escape by, for example, enhancing PD-L1 expression, driving M2 TAM polarization, or suppressing T cell function (Irimie et al., 2017). Inhibiting these oncogenic ncRNAs presents a rational therapeutic strategy, but how to effectively and specifically deliver therapeutic drugs to tumor cells remains a huge challenge.

#### 3.1.1 Antisense oligonucleotides (ASOs)

ASOs are short, synthetic strands of nucleic acid designed to bind with high specificity to a target RNA molecule through Watson-Crick base pairing. This binding can lead to the degradation of the target RNA or sterically block its function (e.g., preventing interaction with proteins or miRNA binding sites) (Zhang et al., 2019). ASOs can be designed to target specific lncRNAs (like MALAT1 or HOTAIR) or circRNAs (Hua et al., 2025). MALAT1 can sponge miR-125b to increase STAT3 expression (Chang and Hu, 2018), miR-101 to influence EZH2 activity (Ding et al., 2021), and miR-224-5p to modulate KDM2A levels in OSCC (Li et al., 2023). ASOs designed to target MALAT1 have entered preclinical and clinical development for various cancers (Neveu et al., 2024). FLM-7523 (formerly FTX-001), an ASO targeting MALAT1, is reported to be ready for clinical investigation (Neveu et al., 2024). However, the clinical translation of ASOs faces challenges, including ensuring efficient *in vivo* delivery to solid tumors like OSCC, minimizing potential off-target effects, and managing possible immunotoxicity. To date, their development for OSCC remains firmly in the preclinical stage (Lv et al., 2025).

#### 3.1.2 Small molecule inhibitors

While directly targeting RNA structures with small molecules is challenging, indirect inhibition is possible. Small molecules can target proteins essential for the biogenesis, processing, or function of specific ncRNAs. Inhibitors of EZH2 (a component of PRC2) could attenuate the downstream effects of HOTAIR-mediated gene silencing (Ahmad et al., 2024), or lead to the de-repression of tumor-suppressive miRNAs like miR-34a in human pancreatic ductal adenocarcinoma (Li et al., 2021), thereby initiating a cascade of anti-tumor and immunomodulatory effects. In OSCC, HOTAIR binds to EZH2 and induces epigenetic silencing by enhancing H3K27me3 modification levels at the E-cadherin promoter region (Wu et al., 2015). This mechanism suggests that EZH2 inhibitors may restore the expression of tumor suppressor genes such as E-cadherin by blocking the formation of the HOTAIR-PRC2 complex, thereby inhibiting tumor invasion and metastasis. Similarly, inhibitors targeting m6A reader proteins like YTHDF2 are being developed; these could potentially stabilize beneficial m6A-modified RNAs or destabilize oncogenic ones (including mRNAs or ncRNAs regulated by YTHDF2), thereby modulating immune responses (Xiao et al., 2024). While the direct regulation of specific ncRNAs by YTHDF2 in OSCC is an emerging field of investigation, lncRNAs themselves can be m6A-modified, and YTHDF2 could therefore influence their function (Ju et al., 2025). The primary limitation of this indirect strategy is specificity, as these protein targets often have broad cellular functions, which can lead to significant systemic toxicity and this approach is still exploratory and preclinical for OSCC.

#### 3.1.3 CRISPR/Cas9-based silencing

The CRISPR/Cas9 system can be adapted for targeted gene silencing without inducing DNA breaks. Using a catalytically inactive or “dead” Cas9 (dCas9) fused to transcriptional repressor domains (e.g., KRAB domain, or histone modifiers like HDAC1 (Liu J. et al., 2021)), guided by specific guide RNAs (gRNAs), allows for the targeted epigenetic silencing of gene promoters (Rajanathadurai et al., 2024). This approach holds promise for achieving durable silencing of specific oncogenic lncRNA genes, such as *MALAT1* or *HOTAIR*, by altering local chromatin structure. As previously discussed, MALAT1 is a key promoter of OSCC proliferation, invasion, and metastasis. Preclinical investigations and theoretical considerations suggest that CRISPR-mediated knockout or CRISPRi-mediated transcriptional repression of MALAT1 could possibly inhibit OSCC progression (Panda et al., 2025). The precision of CRISPRi might be particularly advantageous for abundant lncRNAs like MALAT1, which may have structural roles in addition to gene regulation (Gutschner et al., 2013); reducing its transcript levels via CRISPRi could mitigate oncogenic effects without completely ablating other potential cellular functions, thereby minimizing toxicity. Although CRISPR exhibits high precision in targeted gene knockout, the clinical translation of CRISPR-based therapies faces substantial hurdles. The safe and efficient *in vivo* delivery of the entire CRISPR machinery (e.g., Cas9 protein and guide RNAs) to tumor cells remains a major bottleneck, alongside unresolved concerns about long-term safety and off-target effects (Singh et al., 2025). Therefore, this approach is currently confined to preclinical research for OSCC.

### 3.2 Activation strategies for anti-tumorigenic ncRNAs

Conversely, many ncRNAs, particularly miRNAs like miR-34a, miR-138, and the miR-200 family, function as tumor suppressors, inhibiting proliferation, metastasis, CSC properties, and promoting anti-tumor immunity by targeting oncogenes or immune checkpoints. Their expression is often lost or downregulated during oral cancer progression. Restoring the function of these beneficial ncRNAs is another key therapeutic strategy.

#### 3.2.1 miRNA mimics

These are synthetic double-stranded RNA molecules designed to mimic the function of endogenous mature miRNAs. Once delivered into cells, they are processed and incorporated into the RNA-induced silencing complex (RISC) to silence target mRNAs (Ariyoshi et al., 2015; Kawamata et al., 2009). In OSCC,miR-34a is frequently downregulated; however, restoring its levels using mimics has been demonstrated in preclinical models to inhibit OSCC cell proliferation, induce G1 phase arrest, and suppress metastasis and EMT (Li et al., 2021; Li et al., 2015; Ren et al., 2023). However, due to the lack of safe and efficient delivery systems, the clinical development of miRNA mimics has been challenging. A prominent example is the Phase 1 trial of the miR-34a mimic MRX34, which was halted due to severe immune-related adverse events in patients. This trial failure underscores a critical translational hurdle: the toxicity often associated not with the mimic itself, but with the nanoparticle delivery systems required to protect it from degradation *in vivo* (Hong et al., 2020; Beg et al., 2017).

#### 3.2.2 ncRNA delivery systems

Safe and effective delivery is the cornerstone of ncRNA therapeutics. Viral vectors (e.g., lentivirus, adenovirus) or non-viral vectors (e.g., nanovesicles) (Qi et al., 2022) can be engineered to express specific tumor-suppressive ncRNAs, such as lncRNAs like GAS5 or MEG3, or precursors of beneficial miRNAs (Meng et al., 2021), aiming for sustained expression within target cells. Concurrently, leveraging natural or synthetic delivery vehicles is a major focus. Exosomes, which are natural intercellular messengers, can be engineered to carry specific therapeutic ncRNAs (e.g., lncRNA GAS5) (Gezer et al., 2014; Song et al., 2024). In OSCC, tumor cells naturally secrete exosomes containing specific ncRNA profiles that can influence the TME. The lncRNA MEG3 in OSCC cells has been shown to regulate the content of exosomal miR-421, which in turn promotes angiogenesis in endothelial cells (Huang et al., 2024). Similarly, OSCC-derived exosomes carrying hsa_circ_0069313 can modulate Treg cell function (Zhu M. et al., 2023). This natural role of exosomes in OSCC ncRNA biology provides a strong rationale for their use as therapeutic delivery vehicles. However, significant challenges hinder their clinical use. As discussed in recent studies, key translational hurdles include standardizing isolation methods to ensure purity, improving the stability and efficiency of drug encapsulation, and modifying exosome surfaces for better tumor-targeting specificity (Abd-Rabou et al., 2024). These standardization and manufacturing issues must be resolved before exosomes can be reliably used as therapeutic carriers in OSCC. Furthermore, lipid nanoparticles (LNPs) represent a clinically validated platform for RNA delivery, as demonstrated by mRNA vaccines and siRNA therapeutics (Cui S. et al., 2023). While OSCC-specific clinical data for LNP-ncRNA therapeutics are scarce, preclinical studies in other cancers, such as the delivery of miR-101 using nanorods, have demonstrated increased intracellular ncRNA levels and enhanced therapeutic effects (Xin et al., 2019), highlighting the potential for OSCC. Another promising, though entirely preclinical, strategy involves loading ncRNA therapeutics onto biocompatible scaffolds for controlled, localized release. This approach could be particularly useful for preventing local recurrence after surgery. Recent studies have shown that nanofibrous scaffolds can create a supportive microenvironment for cell-based therapies to repair tissue damage, such as after myocardial infarction (Aglan et al., 2024). This principle could be adapted for OSCC, where a scaffold loaded with miRNA mimics or ASOs could be placed in the tumor bed post-resection to modulate the local immune environment and eliminate residual cancer cells, which may effectively eliminate the recurrence caused by incomplete removal of tumor cells during surgery.

### 3.3 Rationale and potential of combination therapies

Given the complexity of OSCC biology and the multifactorial nature of immune escape and therapy resistance, combination therapies are likely essential for achieving significant clinical benefit. Targeting ncRNAs offers compelling opportunities for synergistic combinations.

#### 3.3.1 Synergistic ncRNA targeting

Combining different ncRNA-based therapies could provide broader pathway coverage. For example, simultaneously inhibiting an oncogenic lncRNA that promotes proliferation (e.g., HOTAIR (154)) and delivering a tumor-suppressive miRNA mimic that enhances apoptosis or immune recognition (e.g., miR-34a (Li et al., 2015)) might yield greater efficacy.

Perhaps the most immediate potential lies in combining ncRNA modulators with ICIs (e.g., anti-PD-1/PD-L1 antibodies) (Kujan et al., 2020). Since ncRNAs regulate checkpoint expression (e.g., MALAT1/miR-200/PD-L1 (Xu et al., 2024), miR-138/PD-1/CTLA-4 (Wei et al., 2016)) and other resistance mechanisms within the TME (e.g., M2 TAM polarization, T cell exhaustion, metabolic barriers) (Zhang B. et al., 2024), targeting these ncRNAs could potentially reverse resistance and re-sensitize tumors to ICI therapy (Zhou et al., 2023). For instance, inhibiting YTHDF2 showed synergistic effects with anti-PD-L1 (166), and delivering miR-138 mimics could enhance immune checkpoint blockade by targeting multiple checkpoints simultaneously (Liu W. et al., 2021).

#### 3.3.2 Combination with conventional therapies

ncRNAs are deeply involved in regulating sensitivity and resistance to chemotherapy and radiotherapy (Gao et al., 2024). Identifying and targeting the specific ncRNAs that mediate resistance to agents commonly used in OSCC (e.g., cisplatin, 5-FU) could significantly improve the efficacy of these standard treatments. For example, targeting lncRNAs like XIST (Liu H. et al., 2022) or H19 (Yue et al., 2021) implicated in chemoresistance could be beneficial.

The rationale for combining ncRNA modulation with ICIs is particularly strong, addressing the critical clinical challenge of immunotherapy resistance (Vianello et al., 2024; Yang et al., 2023). By targeting ncRNAs that regulate pathways critical for ICI function, ncRNA-based therapies present a promising strategy to improve immunotherapy success rates for OSCC.

## 4 Challenges and future directions

Despite the immense potential of ncRNAs as therapeutic targets in oral cancer, significant hurdles remain before these can be effectively translated into clinical practice. Addressing these challenges requires continued innovation in technology, delivery systems, and clinical trial design.

### 4.1 Overcoming technical hurdles

#### 4.1.1 Mapping ncRNA expression and function with precision

The OSCC TME is highly heterogeneous, comprising diverse cell types interacting in complex spatial arrangements (Sutera et al., 2025). Traditional bulk RNA sequencing averages signals across all cells, masking crucial cell-type-specific expression patterns and functions of ncRNAs (Wei et al., 2024). To truly understand how ncRNAs orchestrate immune escape in specific cellular contexts (e.g., ncRNA expression in M2 vs. M1 TAMs, or in exhausted vs. effector T cells), higher resolution techniques are essential. Single-cell RNA sequencing (scRNA-seq) allows for the profiling of ncRNA expression at the individual cell level, revealing cellular heterogeneity (Guo et al., 2024). Spatial transcriptomics (ST) goes a step further by mapping gene (including ncRNA) expression within the intact tissue architecture, providing insights into the spatial organization of cells and their interactions within specific niches or “ecosystems” that influence immune responses and immunotherapy outcomes (Abdulrahman et al., 2025). Applying these technologies to OSCC tissues, particularly before and after immunotherapy, will be critical for identifying context-dependent ncRNA functions and relevant therapeutic targets.

#### 4.1.2 Improving in vivo delivery systems

Efficient and safe delivery of RNA therapeutics to the target cells *in vivo* remains a major bottleneck (Hastings and Krainer, 2023). Naked RNA molecules are rapidly degraded and poorly taken up by cells. While delivery systems like LNPs have achieved clinical success for mRNA vaccines (Meng et al., 2025), optimizing them for ncRNA delivery (ASOs, mimics, CRISPR components) specifically to the OSCC TME presents unique challenges. Key requirements include:(I)Developing strategies to direct nanoparticles specifically to tumor cells or distinct immune cell populations (e.g., TAMs, T cells) within the TME, minimizing off-tumor accumulation (Chen et al., 2024). This might involve incorporating targeting ligands (antibodies, aptamers) onto the nanoparticle surface. (II)Ensuring efficient encapsulation of the ncRNA payload, protection from degradation in circulation, effective cellular uptake, and crucially, escape from endosomes to reach the cytoplasm or nucleus where ncRNAs function (Meng et al., 2025). (III)Minimizing immunogenicity and toxicity associated with the delivery vehicle itself (Schock Vaiani et al., 2025). The clinical trial failure of the miR-34a mimic MRX34 due to severe immune-related adverse events underscores the critical importance of safe delivery platforms (Beg et al., 2017). Ongoing research focuses on optimizing LNP lipid composition (e.g., ionizable lipids, helper lipids, PEG-lipids), particle size, and surface charge to enhance delivery and safety (Meng et al., 2025). Engineered exosomes are also being explored as natural delivery vehicles (Ma et al., 2025).

#### 4.1.3 Functional validation

While transcriptomic studies can identify correlations between ncRNA expression and clinical outcomes or immune phenotypes, rigorous functional validation is essential to establish causality and confirm therapeutic targets. This requires robust *in vitro* and *in vivo* models that accurately recapitulate the OSCC TME. CRISPR/Cas9 gene editing technology is invaluable for precisely deleting or modifying ncRNA genes in relevant cell types (tumor cells, immune cells) within these models to directly assess their functional impact on tumor growth, metastasis, and immune response (Rajanathadurai et al., 2024). Furthermore, a deeper understanding of the relationship between ncRNA sequence, structure, and function is needed to design more effective therapeutic interventions.

### 4.2 Bridging the translational gap

#### 4.2.1 Biomarker development and validation

ncRNAs hold significant promise as biomarkers for OSCC, potentially improving early detection, prognosis, and treatment selection (Balakittnen et al., 2023). Detecting ncRNAs in easily accessible body fluids offers a non-invasive approach. Saliva is particularly significant for OSCC due to its direct contact with the oral cavity (Kalmatte et al., 2023). Studies have identified specific miRNAs (e.g., miR-3928, miR-27b, miR-137), lncRNAs (e.g., MALAT1), and circRNAs (e.g., hsa_circ_0001971) that are differentially expressed in the saliva of OSCC patients compared to healthy controls or individuals with potentially malignant disorders (Das et al., 2025). For instance, salivary hsa_circ_0001971 is significantly upregulated in OSCC patients and its expression is correlated with the clinical TNM stage (P = 0.019), as shown in Table 3 (Zhao SY. et al., 2018). A study combining hsa_circ_0001971 with another circRNA, hsa_circ_0001874, for OSCC diagnosis yielded a high area under the ROC curve (AUC) of 0.922, demonstrating significant diagnostic potential (Zhao SY. et al., 2018). Similarly, salivary miR-27b has been identified as a potential biomarker, with one meta-analysis reporting a pooled sensitivity of 0.76 and specificity of 1.00 for its use in diagnosing Oral Lichen Planus (OLP), a precursor to OSCC (Koopaie et al., 2024). In a study on Head and Neck Squamous Cell Carcinoma (HNSCC), of which OSCC is the most common type, salivary miR-3928 was found to be significantly downregulated in patients. This miRNA showed good discriminatory power with an AUC of 0.74 and its expression was correlated with tumor size (p = 0.02) (Fadhil et al., 2020). Additionally, as a lncRNA significantly upregulated in the saliva of OSCC patients, MALAT1 demonstrated excellent performance in a diagnostic accuracy study, with a sensitivity of 95%, a specificity of 90%, and an AUC of 92.2% (Shalaby et al., 2024). These salivary ncRNAs show potential for early diagnosis and correlation with disease stage (Das et al., 2025). Circulating ncRNAs in blood (plasma or serum) are also being investigated (Ito et al., 2023). However, large-scale, prospective clinical validation studies are crucial to confirm the sensitivity, specificity, and clinical utility of these potential biomarkers before they can be implemented in practice. Moreover, a critical goal is to identify ncRNA signatures that can predict a patient’s response to specific therapies, particularly immunotherapy (Sun and He, 2024). Identifying patients likely to respond or develop resistance to ICIs based on their ncRNA profiles could enable better patient stratification and personalized treatment strategies, avoiding unnecessary toxicity and cost for non-responders.

**TABLE 3:** Examples of ncRNAs with Potential as Non-invasive Biomarkers for OSCC.

ncRNA	Source	Biomarker application	Diagnostic/Prognostic value	References(s)
hsa_circ_0001971	Saliva	Diagnosis, Staging	Combined with hsa_circ_0001874 for diagnosis, the AUC reached 0.922. Expression is correlated with clinical TNM stage	Zhao et al. (2018b)
hsa_circ_0001874	Saliva	Diagnosis	Used in a diagnostic panel with hsa_circ_0001971, yielding a combined AUC of 0.922	Zhao et al. (2018b)
miR-27b	Saliva	Diagnosis (for precursor lesion OLP)	For diagnosing Oral Lichen Planus (OLP), a meta-analysis reported a pooled sensitivity of 0.76 and specificity of 1.00	Koopaie et al. (2024)
miR-3928	Saliva	Diagnosis	Showed discriminatory power for HNSCC with an AUC of 0.74. Its expression was correlated with tumor size	Fadhil et al. (2020)
MALAT1	Saliva	Diagnosis, Prognosis	At a cut-off value of 2.24, exhibited sensitivity of 0.95 and specificity of 0.90 for differentiating OSCC from healthy subjects. The AUC was 0.92	Shalaby et al. (2024)

#### 4.2.2 Clinical trial design

Translating promising preclinical findings into effective therapies requires well-designed clinical trials. The current landscape for OSCC trials often involves small, single-center, non-randomized studies, although immunotherapy trials are increasing (Zeng et al., 2024). Specific interventional trials evaluating ncRNA-based therapeutics for OSCC are still very limited. Future trials must prioritize safety, establish optimal dosing and delivery strategies, and incorporate predictive biomarkers for patient selection (Ito et al., 2023). Learning from past setbacks, like the MRX34 trial, is essential for designing safer and more effective next-generation ncRNA therapies. Collaboration and standardized reporting are needed to advance the field.

#### 4.2.3 Artificial intelligence (AI) and multi-omics integration

The complexity of ncRNA regulatory networks, TME interactions, metabolic influences, and patient heterogeneity necessitates sophisticated analytical approaches (Tao et al., 2025). AI and machine learning (ML) algorithms are powerful tools for integrating large, high-dimensional datasets from multiple “omics” layers (genomics, transcriptomics including ncRNAs, proteomics, metabolomics, spatial omics) with clinical data (Lopes et al., 2025; Singh et al., 2024). This integrated approach can help uncover complex patterns and interactions that are missed by analyzing single data types (Spooner et al., 2025). It can also identify robust ncRNA-based biomarker signatures for diagnosis, prognosis, and prediction of immunotherapy response and enable the development of predictive models to guide personalized treatment decisions (Tao et al., 2025). Additionally, it generates new hypotheses about ncRNA function and regulatory networks within the OSCC TME. The application of AI/ML to multi-omics data, including ncRNA profiles, represents a crucial path forward for realizing the potential of precision oncology in oral cancer.

## 5 Conclusion

Immunotherapy resistance remains a critical barrier to improving outcomes for patients with OSCC. This review has synthesized the compelling evidence establishing ncRNAs—encompassing miRNAs, lncRNAs, and circRNAs—as pivotal regulators orchestrating this resistance. We have detailed how these molecules intricately modulate anti-tumor immunity and tumor biology through diverse and often interconnected mechanisms. These include the fine-tuning of crucial immune checkpoint pathways like PD-1/PD-L1, the induction of T cell exhaustion and promotion of regulatory T cell suppression, the profound remodeling of the tumor microenvironment via metabolic reprogramming and myeloid cell manipulation, and the direct influence on intrinsic tumor cell properties such as epigenetic states and cancer stem cell characteristics.

The deep involvement of ncRNAs in these fundamental processes positions them as highly attractive therapeutic targets and potential biomarkers. Strategies aimed at inhibiting oncogenic ncRNAs or restoring tumor-suppressive ones offer novel avenues to combat OSCC progression and treatment failure. The prospect of combining ncRNA-targeted therapies with existing treatments, particularly immune checkpoint inhibitors, provides a rational and promising approach to synergistically enhance anti-tumor responses and overcome resistance mechanisms.

However, significant hurdles must be overcome to translate this potential into clinical reality. Further research, leveraging advanced single-cell and spatial technologies, is crucial to fully map the complex ncRNA regulatory networks within the heterogeneous OSCC microenvironment. Paramount challenges include the development and optimization of safe, efficient, and precisely targeted delivery systems for ncRNA therapeutics *in vivo*, alongside the rigorous validation of specific ncRNAs as reliable predictive biomarkers for treatment response. Future efforts focused on addressing these challenges through continued mechanistic investigation, technological innovation, and well-designed translational studies will be essential to ultimately harness the power of ncRNA modulation for more effective and personalized therapeutic strategies against oral cancer.
